# Supplementary material for: Online biology degree program broadens access for women, first-generation to college, and low-income students, but grade disparities remain
Source: PLoS One. 2020 Dec 11;15(12):e0243916. doi: 10.1371/journal.pone.0243916 (PMC7732118; doi:10.1371/journal.pone.0243916)
Supplement: S2 File — (DOCX) [file pone.0243916.s002.docx]

[S2 File 1](#_Toc52788425)

[Supplemental Methods 1](#_Toc52788426)

[Results from Alternative Withdraws-Excluded Models 1](#_Toc52788427)

[Other Supplemental Tables 6](#_Toc52788428)

[Complete Data and Analysis Code 13](#_Toc52788430)

# Supplemental Methods

The alternative model (U) is identical to model B, but uses a “priorGPA” variable in place of GPAO (S3 Table). Here priorGPA is a composite of high school GPA and transfer GPA. Among in-person students, only 18% have missing high school GPA compared to 72% missing among online students. In contrast, only 13% of online students have missing transfer GPA. Therefore, priorGPA is set to the high school GPA for all in-person students and to transfer GPA for all online students.

# Results from Alternative Withdraws-Excluded Models

The models presented in this section are specified identically to the models in the main text, but they have been run using a dataset with withdraw grades removed rather than being treated as failures as they were in the main text. We will describe the major differences between these models and the main text models below. One notable difference throughout is that the negative grade effect associated with online instruction is smaller in these models. This is because the online students withdraw at greater rates than the in-person students, thus the models in which withdraws are treated as failures necessarily show a greater penalty (see Table 1, main text).

***Finding 2_non-W_: Women, first-generation, and BLNP students receive lower grades regardless of modality. Pell status has no significant effect. For BLNP students, grade deficits are reduced in online courses.***

Table S1. Withdraws-excluded models compared for research question 2

| A*_non-W_*: Course Grade ~ GPAO + Gender + College Generation + Pell Eligibility + Race/Ethnicity +  (1 \| STUDENT) + (1 \| SECTION)  B*_non-W_*: ~ {Model A} + Online  C*_non-W_*: ~ {Model B} + Gender : Online  D*_non-W_*: ~ {Model B} + College Generation : Online  E*_non-W_*: ~ {Model B} + Pell Eligibility : Online  F*_non-W_*: ~ {Model B} + Race/Ethnicity : Online |
| --- |

As in the main text, model B_non-w_ significantly improved model fit (likelihood ratio test, *p* < .001) and showed that the online mode of instruction was associated with a large, negative grade difference. This difference is smaller than what was seen with withdraw grades included. Whereas model D_non-w_, which includes an interaction between first-generation status and mode of instruction, was the best-fitting model in the main text, model F_non-w_, which instead includes an interaction between race/ethnicity and mode of instruction was the best fit when withdraws are excluded (S2 Table).

Table S2. Withdraws-excluded regression results for research questions 2a and 2b. BLNP refers to Black, Latinx, Native American, and Pacific Islanders. First-generation students are those for whom neither parent had earned a college degree. Pell eligible refers to low-income students.

|  | Model A_non-w_ | Model B_non-w_ | Model F_non-w_ |
| --- | --- | --- | --- |
| Intercept | 2.964***  (0.029) | 3.003***  (0.029) | 3.019***  (0.030) |
| GPAO | 0.837***  (0.013) | 0.838***  (0.013) | 0.839***  (0.013) |
| Gender (W) | -0.095***  (0.023) | -0.089***  (0.023) | -0.090***  (0.023) |
| College Generation (FG) | -0.089***  (0.024) | -0.086***  (0.024) | -0.083***  (0.024) |
| Pell Eligibility (Y) | -0.032  (0.023) | -0.029  (0.023) | -0.027  (0.023) |
| Race/Ethnicity (BLNP) | -0.140***  (0.024) | -0.140***  (0.024) | -0.190***  (0.029) |
| Online (Y) |  | -0.374***  (0.065) | -0.424***  (0.067) |
| Online (Y) : Race/Ethnicity (BLNP) |  |  | 0.142**  (0.048) |
| AIC  R-squared_marginal_  No. Observations | 22070  0.373  9175 | 22039  0.392  9175 | 22032  0.393  9175 |

Standard errors are reported in parentheses.

*, **, *** indicate significance at the 95%, 99%, and 99.9% levels, respectively.

***Finding 3_non-W_: Students who are both Pell eligible and identify as BLNP receive lower grades.***

Beginning from the initial, no-interaction model from the previous section (model A_non-w_), we tested additional pairwise interactions between the demographic terms (G_non-w_–L_non-w_, S3 Table). Of these, model G_non-w_ has the most support from AIC and significantly improved model fit (likelihood ratio test, *p* = .005), offering evidence that BLNP students who are Pell eligible receive lower grades than BLNP students who are not Pell eligible (S4 Table). This model was also the best fitting in the main text, although in that analysis, it was not a statistically significant improvement over model A_non-w_.

Table S3. Withdraws-excluded models compared for research question 3

| A*_non-W_*: Course Grade ~ GPAO + Gender + College Generation + Pell Eligibility + Race/Ethnicity +  (1 \| STUDENT) + (1 \| SECTION)  G*_non-W_*: ~ {Model A} + Pell Eligibility : Race/Ethnicity  H*_non-W_*: ~ {Model A} + College Generation : Race/Ethnicity  I*_non-W_*: ~ {Model A} + Gender : Race/Ethnicity  J*_non-W_*: ~ {Model A} + College Generation : Pell Eligibility  K*_non-W_*: ~ {Model A} + Gender : College Generation  L*_non-W_*: ~ {Model A} + Gender : Pell Eligibility |
| --- |

Table S4. Withdraws-excluded regression results for research question 3. BLNP refers to Black, Latinx, Native American, and Pacific Islanders. First-generation students are those for whom neither parent had earned a college degree. Pell eligible refers to low-income students.

|  | Model A_non-w_ | Model G_non-w_ |
| --- | --- | --- |
| Intercept | 2.964***  (0.029) | 2.945***  (0.029) |
| GPAO | 0.837***  (0.013) | 0.838***  (0.013) |
| Gender (W) | -0.095***  (0.023) | -0.093***  (0.023) |
| College Generation (FG) | -0.089***  (0.024) | -0.088***  (0.024) |
| Pell Eligibility (Y) | -0.032  (0.023) | 0.011  (0.028) |
| Race/Ethnicity (BLNP) | -0.140***  (0.024) | -0.070*  (0.034) |
| Pell Eligibility (Y) : Race/Ethnicity (BLNP) |  | -0.130**  (0.046) |
| AIC  R-squared_marginal_  No. Observations | 22070  0.373  9175 | 22064  0.374  9175 |

Standard errors are reported in parentheses.

†, *, **, *** indicate significance at the 90%, 95%, 99%, and 99.9% levels, respectively.

***Finding 4_non-W_: The patterns of differential grade impacts for students holding multiple identities underrepresented in STEM do not differ when mode of instruction is considered.***

Using model F_non-w_ as a point of comparison, we again tested the inclusion of all possible pairwise interactions for the demographic categories, but here we also included an interaction between these pairwise combinations and instruction mode (S5 Table). Of these, the best fitting was model M_non-w_, which includes a three-way “Pell eligibility : race/ethnicity : instruction model” interaction (S6 Table), and significantly improved the model fit over model F_non-w_ (likelihood ratio test, *p* = .01). This follows directly from the best fitting models from the prior sections, models F_non-w_ and G_non-w_. However, the results in Table S6 show that the three-way interaction term itself is non-significant. The model can be reduced to a simple combination of the terms in models F_non-w_ and G_non-w_ (race/ethnicity : online interaction and Pell eligibility : race/ethnicity). Results of this model (altM_non-w_) are also shown in S6 Table.

Table S5. Withdraws-excluded models compared for research question 4

| B*_non-W_*: Course Grade ~ GPAO + Online + Gender + College Generation + Pell Eligibility +  Race/Ethnicity + (1 \| STUDENT) + (1 \| SECTION)  F*_non-W_*: ~ {Model B} + Race/Ethnicity : Online  M*_non-W_*: ~ {Model B} + Pell Eligibility : Race/Ethnicity : Online  N*_non-W_*: ~ {Model B} + College Generation : Race/Ethnicity : Online  O*_non-W_*: ~ {Model B} + Gender : Race/Ethnicity : Online  P*_non-W_*: ~ {Model B} + College Generation : Pell Eligibility : Online  Q*_non-W_*: ~ {Model B} + Gender : College Generation : Online  R*_non-W_*: ~ {Model B} + Gender : Pell Eligibility : Online  altM*_non-W_*: ~ {Model B} + Pell Eligibility : Race/Ethnicity + Race/Ethnicity : Online |
| --- |

Table S6. Withdraws-excluded regression results for models examining whether online modality exacerbates or reduces any grade disparities for students holding multiple social identities underrepresented in STEM. BLNP refers to Black, Latinx, Native American, and Pacific Islanders. First-generation students are those for whom neither parent had earned a college degree. Pell eligible refers to low-income students.

|  | Model F_non-w_ | Model M_non-w_ | Model altM_non-w_ |
| --- | --- | --- | --- |
| Intercept | 3.019***  (0.030) | 3.003***  (0.031) | 3.000***  (0.030) |
| GPAO | 0.839***  (0.013) | 0.840***  (0.023) | 0.840***  (0.013) |
| Gender (W) | -0.090***  (0.023) | -0.088***  (0.023) | -0.089***  (0.023) |
| College Generation (FG) | -0.083***  (0.024) | -0.083***  (0.024) | -0.081***  (0.024) |
| Pell Eligibility (Y) | -0.027  (0.023) | 0.013  (0.034) | 0.022  (0.028) |
| Race/Ethnicity (BLNP) | -0.190***  (0.029) | -0.134**  (0.041) | -0.116**  (0.037) |
| Online (Y) | -0.424***  (0.067) | -0.442***  (0.072) | -0.430***  (0.067) |
| Online (Y) : Race/Ethnicity (BLNP) | 0.142**  (0.048) | 0.206**  (0.073) | 0.152**  (0.048) |
| Online (Y) :  Pell Eligibility (Y) |  | 0.025  (0.056) |  |
| Pell Eligibility (Y) :  Race/Ethnicity (BLNP) |  | -0.111†  (0.058) | -0.144**  (0.046) |
| Online (Y) :  Pell Eligibility (Y) :  Race/Ethnicity (BLNP) |  | -0.097  (0.097) |  |
| AIC  R-squared_marginal_  No. Observations | 22032  0.393  9175 | 22028  0.395  9175 | 22025  0.395  9175 |

Standard errors are reported in parentheses.

†, *, **, *** indicate significance at the 90%, 95%, 99%, and 99.9% levels, respectively.

# Other Supplemental Tables

Table S7. Student racial/ethnic demographics prior to isolating white and BLNP (Black, Latinx, Native American, and Pacific Islander) students

| Race/Ethnic Group | Online | In-Person |
| --- | --- | --- |
| Asian | 164 | 1233 |
| White | 2027 | 4723 |
| Two or More | 209 | 442 |
| Other | 20 | 62 |
| BLNP (total) | 1048 | 2451 |
| *Black or African American* | *278* | *407* |
| *Hispanic* | *700* | *1916* |
| *American Indian or Alaska Native* | *54* | *110* |
| *Native Hawaiian or Pacific Islander* | *16* | *18* |

Table S8. Fit statistics for main models

| Model | Research Question | df | AIC | BIC | Log Likelihood |
| --- | --- | --- | --- | --- | --- |
| A | 2, 3 | 9 | 30058 | 30123 | -15020 |
| B | 2 | 10 | 30019 | 30091 | -14999 |
| C | 2 | 11 | 30019 | 30099 | -14999 |
| D | 2, 4 | 11 | 30014 | 30094 | -14996 |
| E | 2 | 11 | 30020 | 30100 | -14999 |
| F | 2 | 11 | 30018 | 30098 | -14998 |
| G | 3 | 10 | 30056 | 30128 | -15018 |
| H | 3 | 10 | 30058 | 30131 | -15019 |
| I | 3 | 10 | 30060 | 30132 | -15020 |
| J | 3 | 10 | 30059 | 30131 | -15019 |
| K | 3 | 10 | 30059 | 30132 | -15020 |
| L | 3 | 10 | 30057 | 30130 | -15019 |
| M | 4 | 14 | 30019 | 30120 | -14996 |
| N | 4 | 14 | 30017 | 30118 | -14994 |
| O | 4 | 14 | 30022 | 30124 | -14997 |
| P | 4 | 14 | 30019 | 30120 | -14996 |
| Q | 4 | 14 | 30015 | 30116 | -14993 |
| R | 4 | 14 | 30020 | 30121 | -14996 |

Table S9. Fit statistics for withdraws-excluded models

| Model | Research Question | df | AIC | BIC | Log Likelihood |
| --- | --- | --- | --- | --- | --- |
| A_non-w_ | 2, 3 | 9 | 22070 | 22123 | -11026 |
| B_non-w_ | 2 | 10 | 22039 | 22110 | -11010 |
| C_non-w_ | 2 | 11 | 22041 | 22119 | -11010 |
| D_non-w_ | 2 | 11 | 22037 | 22116 | -11008 |
| E_non-w_ | 2 | 11 | 22041 | 22119 | -11010 |
| F_non-w_ | 2, 4 | 11 | 22032 | 22111 | -11005 |
| G_non-w_ | 3 | 10 | 22064 | 22136 | -11022 |
| H_non-w_ | 3 | 10 | 22068 | 22139 | -11024 |
| I_non-w_ | 3 | 10 | 22072 | 22143 | -11026 |
| J_non-w_ | 3 | 10 | 22072 | 22143 | -11026 |
| K_non-w_ | 3 | 10 | 22072 | 22143 | -11026 |
| L_non-w_ | 3 | 10 | 22071 | 22142 | -11025 |
| M_non-w_ | 4 | 14 | 22028 | 22128 | -11000 |
| N_non-w_ | 4 | 14 | 22031 | 22131 | -11002 |
| O_non-w_ | 4 | 14 | 22037 | 22137 | -11005 |
| P_non-w_ | 4 | 14 | 22043 | 22142 | -11007 |
| Q_non-w_ | 4 | 14 | 22042 | 22142 | -11007 |
| R_non-w_ | 4 | 14 | 22044 | 22144 | -11008 |
| altM_non-w_ | 4 | 12 | 22025 | 22110 | -11000 |

Table S10. Regression results for research question 3. BLNP refers to Black, Latinx, Native American, and Pacific Islanders. First-generation students are those for whom neither parent had earned a college degree. Pell eligible refers to low-income students.

|  | Model A | Model G |
| --- | --- | --- |
| Intercept | 2.664***  (0.038) | 2.646***  (0.040) |
| GPAO | 0.643***  (0.015) | 0.644***  (0.015) |
| Gender (W) | -0.078*  (0.032) | -0.076*  (0.032) |
| College Generation (FG) | -0.085*  (0.033) | -0.085*  (0.033) |
| Pell Eligibility (Y) | -0.109***  (0.032) | -0.067†  (0.039) |
| Race/Ethnicity (BLNP) | -0.215***  (0.034) | -0.148**  (0.049) |
| Pell Eligibility (Y) : Race/Ethnicity (BLNP) |  | -0.124†  (0.066) |
| AIC  R-squared_marginal_  No. Observations | 30058  0.215  10249 | 30056  0.215  10249 |

Standard errors are reported in parentheses.

†, *, **, *** indicate significance at the 90%, 95%, 99%, and 99.9% levels, respectively.

Table S11. Model specifications for alternative models

| S^1^: Course Grade ~ GPAO_sgc_ + Gender + College Generation * Online + Pell Eligibility +  Race/Ethnicity + (1 \| STUDENT) + (1 \| SECTION)  T^1^: ~ {Model S} + Gender : College Generation : Online |
| --- |
| U^2^: Course Grade ~ priorGPA + Online + Gender + College Generation + Pell Eligibility +  Race/Ethnicity + (1 \| STUDENT) + (1 \| SECTION) |

^1^Subgroup-centered

^2^Prior GPA

Table S12. Regression results for alternative subgroup-centered models examining the degree to which the main models underestimate the true demographics differences. BLNP refers to Black, Latinx, Native American, and Pacific Islanders. First-generation students are those for whom neither parent had earned a college degree. Pell eligible refers to low-income students.

|  | Model D | Model S  subgroup-centered | Model Q | Model T subgroup-centered |
| --- | --- | --- | --- | --- |
| Intercept | 2.740***  (0.040) | 2.858***  (0.040) | 2.697***  (0.045) | 2.771***  (0.045) |
| GPAO | 0.643***  (0.015) | 0.637***  (0.015) | 0.641***  (0.015) | 0.640***  (0.015) |
| Gender (W) | -0.064*  (0.032) | -0.087**  (0.033) | 0.011  (0.049) | 0.066  (0.049) |
| College Generation (FG) | -0.151***  (0.043) | -0.268***  (0.043) | -0.074  (0.065) | -0.124†  (0.066) |
| Pell Eligibility (Y) | -0.095**  (0.032) | -0.138***  (0.033) | -0.093**  (0.032) | -0.133***  (0.033) |
| Race/Ethnicity (BLNP) | -0.212***  (0.033) | -0.393***  (0.033) | -0.210***  (0.033) | -0.389***  (0.033) |
| Online (Y) | -0.571***  (0.082) | -0.584***  (0.082) | -0.454***  (0.101) | -0.340***  (0.102) |
| Online (Y) :  College Generation (FG) | 0.170**  (0.065) | 0.278***  (0.065) | 0.018  (0.118) | -0.016  (0.118) |
| Online (Y) :  Gender (W) |  |  | -0.178*  (0.089) | -0.372***  (0.089) |
| College Generation (FG) :  Gender (W) |  |  | -0.133  (0.083) | -0.252**  (0.083) |
| Online (Y) :  College Generation (FG) :  Gender (W) |  |  | 0.235†  (0.141) | 0.454**  (0.141) |
| AIC  R-squared_marginal_  No. Observations | 30014  0.242  10249 | 30047  0.238  10249 | 30015  0.243  10249 | 30032  0.241  10249 |

Standard errors are reported in parentheses.

†, *, **, *** indicate significance at the 90%, 95%, 99%, and 99.9% levels, respectively.

Table S13. Regression results for alternative prior GPA model examining the degree to which the use of college GPA data in the main models reduces demographic effects. BLNP refers to Black, Latinx, Native American, and Pacific Islanders. First-generation students are those for whom neither parent had earned a college degree. Pell eligible refers to low-income students.

|  | Model B | Model U  Prior GPA |
| --- | --- | --- |
| Intercept | 2.721***  (0.039) | 2.780***  (0.052) |
| GPAO | 0.644***  (0.015) |  |
| priorGPA |  | 0.716***  (0.037) |
| Gender (W) | -0.066*  (0.032) | -0.171***  (0.047) |
| College Generation (FG) | -0.081*  (0.033) | -0.138**  (0.048) |
| Pell Eligibility (Y) | -0.104**  (0.032) | -0.078†  (0.046) |
| Race/Ethnicity (BLNP) | -0.215***  (0.032) | -0.319***  (0.048) |
| Online (Y) | -0.500***  (0.078) | -0.475***  (0.085) |
| R-squared_marginal_  No. Observations | 0.241  10249 | 0.127  8508 |

Standard errors are reported in parentheses.

†, *, **, *** indicate significance at the 90%, 95%, 99%, and 99.9% levels, respectively.

# Complete Data and Analysis Code

The anonymous data and R code sufficient to reproduce the analyses in this study are included in the supplemental materials. Four files are included in a zip archive. The models in the main text are found in “analysis file.Rmd” and are based on “gradesData.csv”. The withdraws-excluded models are found in “analysis file (no W).Rmd” and are based on “gradesData (no W).csv”.
