# Supplementary material for: Online biology degree program broadens access for women, first-generation to college, and low-income students, but grade disparities remain
Source: PLoS One. 2020 Dec 11;15(12):e0243916. doi: 10.1371/journal.pone.0243916 (PMC7732118; doi:10.1371/journal.pone.0243916)
Supplement: Attachment — Submitted filename: Response to Reviewers.docx [file pone.0243916.s007.docx]

Dr. Prunuske,

We thank you and the two reviewers for the careful review and helpful comments that have made our manuscript better. We have made all of the suggested changes except for one. We have prepared a detailed response to the comments below, with our comments in red. We would like to explain our one point of disagreement with the reviewers here, because both reviewers had a similar request.

Both reviewers questioned our decision to treat withdraw grades as failures in our regression models, and reviewer #2 argued that an alternative analysis with withdraws excluded completely should be placed in the main text instead. Although it is true that withdraws do not carry the same penalty as failures in GPA calculations, because our analysis focuses on courses that are required to progress in the degree program, we still feel strongly that failure and course withdrawal are both negative outcomes for these students. There is other literature that treats withdrawals as failures for similar reasons (e.g. Freeman et al. 2014 examining DFW rates between active learning and traditional lecturing). That being said, some readers are likely to raise the same question as the reviewers have, so we have added a complete analysis of the data with withdraws removed in the Supplemental Materials. This analysis found many of the same effects as our main analysis as well as some differences. We reference this analysis in the main text (lines 266, 395, 682–692). This analysis was already alluded to in the prior version, but this can now be linked with the full detailed results. Related to this topic is the fact that withdraw rates vary systematically between the online courses and the in-person courses. As part of our revisions, we have added a new table to the main text presenting the withdraw rates by demographic groups for both online and in-person courses. This will help readers understand the context of our data and why both options with respect to the treatment of withdraws introduce their own biases in our results. We agree with reviewer 1 that students might withdraw from a course for a multitude of reasons that are not related to their course performance. However, the demographic differences in withdrawal rates in the in-person and online program are concerning and we think that removing withdraws altogether from our analyses would work to obscure inequities. We hope that the inclusion of this new analysis in full in the Supplement and the expanded discussion of the withdraws decision and its consequences in the main text will be an acceptable compromise to the reviewers.

Sincerely,

Chris Mead & K. Supriya (co-first authors)

Yi Zheng

Ariel D. Anbar

James P. Collins

Paul LePore

Sara E. Brownell

Additional Editor Comments (if provided):

The authors complete an important analysis comparing outcomes between online and face-to-face instruction comparing different student identities. The reviewers are favorable about the authors' analysis and offer several suggestions to improve the manuscript. One area that I would urge the authors to address is a better description of the online vs in person course elements that might be contributing to these outcomes. It is critical that biology educators understand what practices are leaving some students behind. For example consider the work of Joe Feldman Grading for Equity. I look forward to seeing a revised version of this work.

Thank you. We certainly agree that grading and assessment decisions play a huge role in equity in education and we know that instructors of similar courses can make very different assessment decisions. Detailed information on these choices within the studied courses was not readily available to us and outside of the scope of our research questions. It is important that readers understand this as well, so we have expanded our remarks in the Methods section to make this clear (line 252). However, this would be an interesting area of future research.

Reviewers' comments:

Reviewer #1: The authors use rigorous statistical analysis to compare the ability of a fully-online biology degree program to improve access and grade disparities for groups traditionally underrepresented the life sciences. The authors are uniquely positioned to undertake such a study, having access to Registrar data at Arizona State University, one of the only universities in which parallel fully-online and in-person biology degree programs are available. Overall, the authors have done a good job at presenting a thorough analysis to support their conclusions of the ability of the fully-online curriculum to address traditional concerns to equity and inclusion in life science education. The following are questions and suggestions that will hopefully help the clarity and overall strength of the manuscript:

-Throughout the manuscript, the authors cite the fact that students in online courses tend to get lower grades than their in-person counterparts and that such disparities do not, in large part, seem to be corrected by the fully-online curriculum. Left unsaid are the assessment techniques that are used in online versus in-person courses and the instructors who are doing the evaluating the students in each modality. Clearly, these are considerations that have been looked at in other studies and are not the direct subject of this work. However, it would be helpful when considering the reasons behind grade disparities to have a better sense of the role of particular assessment tools in generating these disparities. In short, how is grading done in each modality, who is doing the grading, and how do these contribute to the disparities on which this paper is focused?

We agree with the reviewer that assessments would impact course grades. Detailed information on these choices within the studied courses is not readily available to us, nor was it one of our research questions. However, we have no indication that there are systematic differences among online and in person courses. In fact, the online curriculum has been developed to parallel the in-person curriculum in terms of course goals and content. Many of the online courses have also been developed by the same instructor as the in-person course, so they have used similar assessments. It is important that readers understand that while assessments are important, they are outside the scope of this study, so we have expanded our remarks in the Methods section to make this clear (line 252). We have also added some sentences about how grading practices affect grade disparities in the discussion (lines 542) and how this might be included in future work (line 712).

-The authors reference the fact that online course are offered in 7.5-week sessions as opposed to the 15-week offerings of the in-person format. This would suggest an accelerated pacing to the online courses that could play a significant role in the grade disparities that are observed between what is seen in the two modalities. It would be good to hear more about the author's perspective on the potential role of course pacing on student performance.

We thank the reviewer for this point. We do agree that it’s a possible explanation for some of the grade differences. We have added a remark at line 249 to say that the overall semester load is equivalent. We also now discuss this in the Discussion at line 499.

-The authors choose to treat students who withdraw from the courses as having failed the courses and provide extensive rationale for their choice while acknowledging potential limitations to their analysis. My concern stems from the fact that withdrawal from a course can be the result of several unpredictable pressures on a student's life that have nothing to do with poor course performance. Would simply removing withdrawing students from the data set eliminate some of the analytical problems that this cohort introduces to the study?

Please see the response provided at the top.

-In Table 1, expressing the results as percentages in an additional column would make the results easier to digest.

Table 1 has now been removed entirely because it was duplicative of Figure 1.

-In Tables 2, 4, and 5, it is difficult to follow the progression of the models presented by what is in the Table itself. For each, a Legend that highlights the differences between each model would be appreciated. This would be redundant to the text of the paper but make the interpretation of each Table as one reads the manuscript markedly easier.

We agree that the tables are difficult to read in the prior format. We have rewritten these tables so that they present only the newly added predictors for each model.

-On a related note, in several spots in the text (lines 339-45, 510-13, 545, 551-2), the authors present their Models solely by their single letter identification. It is difficult to recall during the reading which models are which. For clarity, adding the feature of the model in parentheses after the model name would be appreciated.

Thank you for this suggestion. We have added some text to those sections to improve the readability. We also hope that our changes in response to the previous comment will make it easier for readers to glance at the relevant table to quickly see what each model’s distinctive features are.

-Lines 357-65 and 410-418 appear to be the Figure Legends for Figures 2 and 3 embedded into the text. These, clearly, will need to be moved to the actual location in which these Figures will be placed (Disregard if this placement was done at the direction of the journal).

Yes, this was part of the journal formatting requirement for separating the figures from the main text.

-Lines 51-53 introduce the concept of the "education desert". Some additional language for why these deserts exist would help contextualize the argument set-up here.

We have added some more details about the concept of the education desert (lines 51-62)

-In lines 158-64, the authors provide citations describing improved confidence and personal value in science amongst students taking online classes. Some additional language describing why this is so would help contextualize the argument being set-up here.

Thank you for this suggestion. Improved confidence in career choice and personal value in science for online students likely emerges from the fact that they are often older and have more work experience. We have added this in line 182 now.

-Line 231: place "white" in parentheses.

Thank you, yes, this was a typo.

I hope that these comments will help to produce a final paper that more effectively conveys its important messages.

Reviewer #2: Overview

This paper describes the analysis and interpretation of student achievement as well as demographic and socioeconomic data from an institution of higher education’s registrar’s office. It provides insights into student achievement in an online and face-to-face biology degree program based on students’ “social groups”. The manuscript is well-organized, with clear research questions and a results and discussion section formatted around those questions, and well written. It contains important and novel results that would be of interest to biology educators and program administrators and thus merits publication with some revisions.

Suggestions for improvement or changes

Introduction

The introduction thoroughly demonstrates the potential for online courses to provide access and promote diversity in science. It also reviews literature indicating that students often perform worse in online courses and demonstrates that the data analyzed in the manuscript are unique in that they are results from students enrolled in an online or face-to-face STEM program.

However, the introduction does not describe what is known about how students in the social groups examined typically perform in a face-to-face classroom. Since the title mentions grade gaps remaining, it would be good to establish what is known about the grade gaps in face-to-face courses for these groups.

We thank the reviewer for bringing this to our attention. While we did talk about the disparities in in-person courses in the context of studies that compared in-person and online courses, we did not include anything about the broader in-person literature in the introduction. We have now added a sentence and references about it (lines 151, 192). We have also made some more changes to this paragraph to make our arguments easier to follow.

The term grade gaps in general is central to 3 out of 4 research questions asked and needs to be defined and examined more thoroughly in the introduction. I also want to warn the authors that this reference to “gaps” can be confusing. For example, on line 481 the authors cite an article that describes some reasoning for gender gaps in academic performance. When stated like this it is not clear which gender is expected to perform better and which is expected to perform worse. I felt the same confusion various times with the term in reference to the authors’ own results (i.e. lines 367,379-381). The language in the abstract with reference to higher or lower grades is more precise.

We thank the reviewer for pointing this out. We have added an explanation of what we mean by grade gaps in lines 215-219. We have also edited the language at places where the direction of the difference in grades might not have been clear earlier to make it more explicit.

Methods

The first sentence of the methods is confusing. The authors used data from students enrolled in both fully online and face-to-face biology programs.

Thank you, yes, the previous statement was incorrect. We have revised it to correctly describe the study population.

I disagree with the authors’ decision to score withdraw grades as Fs when calculating the students’ GPAs and this is my basis for answering "no" to question 2. That is not how GPAs are calculated. I did see the note in the discussion about the completion of an analysis that did not score Ws as Fs in the GPA calculation and believe that these are the data that should be presented in the paper. It is well known that withdraw rates are higher in online courses. To count them as Fs is likely having a larger impact on your measure of student achievement (GPA) for online students. Additionally, an earned F from a student who continued to invest time into a course is a different very outcome than choosing to withdraw from a course. Finally, the differences in the withdraw rates by course modality and social groups could be meaningful and may warrant further analysis and interpretation.

Please see the response provided at the top.

I understand the rationale provided in the paper that students who receive a W in a course are not making progress toward their degree and that is an important consideration. Perhaps it deserves another type of analysis that looks at which students are successful (this definition varies, but I typically see that defined as earning grades A-C) compared with those who are not (a category which would include the students who withdrew and received failing grades).

This is partially related to our discussion at the top related to W grades. To this point specifically, although we do agree that a separate analysis of such a binomial outcome (ABC vs DFW) would be interesting, it is not the outcome we are primarily interested in to answer our research questions. The reason for this is the importance among biology students (even more than other science majors) of having a high GPA to be competitive for medical school or other post-graduate opportunities, so we prefer to model it as a continuous rather than binomial outcome.

Results

In general, the results section is focused on the results of the statistical tests. It would improve the clarity of the paper if the authors also included descriptions of the data. For example, the written description of the results in Table 1 is very brief. It could be improved by describing the proportions or differences between the proportions for the groups of students. The same is true for the data demonstrating grade gaps. There is a lot written about the models, but not about the differences in the calculated GPAs for the groups of students.

We have made a number of changes in response to these comments. The results section now begins with the “Finding 1” statement to immediately orient the reader to the significant demographic differences between in-person and online populations. We then provide a description of the data before moving to the statistical analysis.

In the remainder of the Results section, we have inserted new text quoting specific values in the model outputs from the results tables and making explicit the features of models (rather than only using the model abbreviations).

Discussion

There is an incongruence between the results and the interpretation of the results in the discussion. This was my reasoning for answering "partly" to question 1. For example, the authors found similar grade gaps for many students who are underrepresented in the STEM fields regardless of the modality and clearly state that the online format did not reduce grade gaps (line 440). However, some of the studies used to interpret these finding are explanations for why greater gaps may exist in online courses (i.e. line 467-471). The interpretation makes it sound like your data confirmed larger grade gaps in the online format. In general, I recommend a more thorough interpretation of the findings that BOTH modalities are leading to grade gaps for groups underrepresented in STEM.

Thank you for this observation. The reference to Wladis here (formerly line 467, now line 539) was confusing and we have modified it. We have also elaborated slightly on our concluding sentence in this paragraph (line 551).

Additionally in the second paragraph under the “Explanations” heading, I also sense an incongruence between the results and interpretation. This paragraph is interpreting the finding that grade gaps were larger for women in the online classroom. Yet the evidence provided in lines 479-494 is not specific to the online environment.

Thank you for this suggestion. We have made some changes to the phrasing of this paragraph to make clear when and why we are referring to prior work from online or in-person courses.

The discussion could benefit from emphasizing the need for online and face-to-face biology educators to reflect on how their practices are leaving some of their students behind. This message may be even more important for online educators since they likely have more students from social groups that are underrepresented in STEM fields. There is some of this language in the last two paragraphs of the discussion, but the paper may have broader appeal if it provided some resources on best practices for creating an inclusive environment that helps all students learn in online and face-to-face classrooms.

We do agree that this will be a point of interest for some readers, but we are also reluctant to speak too authoritatively on things that are not derived from our study data. We have added a Future Directions section (line 707) to explain to readers what we consider to be the necessary next steps.
