# Supplementary material for: Online biology degree program broadens access for women, first-generation to college, and low-income students, but grade disparities remain
Source: PLoS One. 2020 Dec 11;15(12):e0243916. doi: 10.1371/journal.pone.0243916 (PMC7732118; doi:10.1371/journal.pone.0243916)
Supplement: Attachment — Submitted filename: Response to Reviewers - 2.docx [file pone.0243916.s008.docx]

Reviewer #3: In summary, the manuscript addresses a key research area comparing the access and equity as it relates to online vs in-person instruction. The previous review has resulted in significantly addressing a few areas of concern and hence, improving the overall readability of this work. I am also in agreement with the authors about the concern raised by the previous reviews as it relates to considering the DFW rates as failures in the regression model. The authors address it really well.

I am recommending some minor revisions based upon my reading and they are summarized as follows:

-Introduction: If any references related to existing grade disparities could be reported in the context of “intersectionality”? Cite references related to “Black Feminist Theory” as it relates to biology education studies. Not sure if the authors are pursuing this for the very first time in the given context and hence, "novel".

This is a very good suggestion. We now end this paragraph with a specific example from undergraduate biology of an intersectional effect. This complements our existing broad survey of the importance of intersectionality.

-Add a marker at the bottom of the X-axis to indicate the axis does not start at 0 (or whatever the lowest GPA recorded is-- even if it not 0 , it is most likely not 1.6 and therefore should have a small mark to indicate this) – please see figures 2 and 3.

We have added “minor” tick marks to both figures to make clear that the top and bottom borders are aligned to the labeled portion of the scale. We acknowledge that this is not precisely what the reviewer suggested, but we believe it satisfies the spirit of the suggestion (making the absolute range of the figures explicit).

-Add a citation/weblink from NSF to support line 294

We have added a reference to the most recent NSF special report on Women, Minorities, and Persons with Disabilities in Science and Engineering, which defines “underrepresented minorities” in the way suggest on line 294.

-Recommend tabling models and regressions results – see lines 434, 466, 469

We believe that combining the model specification tables and results tables would be confusing. As such, we will leave these unchanged.

-Recommend adding some elaboration on how results are consistent with some but not all prior reported work – line 496

This is another good suggestion. We now end this paragraph with an elaboration of how the findings in Cavanaugh & Jacquemin (of no online:face to face difference) relate to one of the limitations that we discuss in that paragraph.

Besides, these recommendations, I do not find any inconsistencies in the overall manuscript. It is very well written and would recommend it for publication.
